# Supplementary material for: Targeting the DNA damage repair protein RAD51 alters fibroblast metabolism and enhances apoptosis in pulmonary fibrosis
Source: bioRxiv. 2026 Apr 4:2026.04.01.715935. Preprint. [Version 1] doi: 10.64898/2026.04.01.715935 (PMC13060284; doi:10.64898/2026.04.01.715935)
Supplement: Supplement 1 [file media-1.pdf]

Supplementary Figure S1

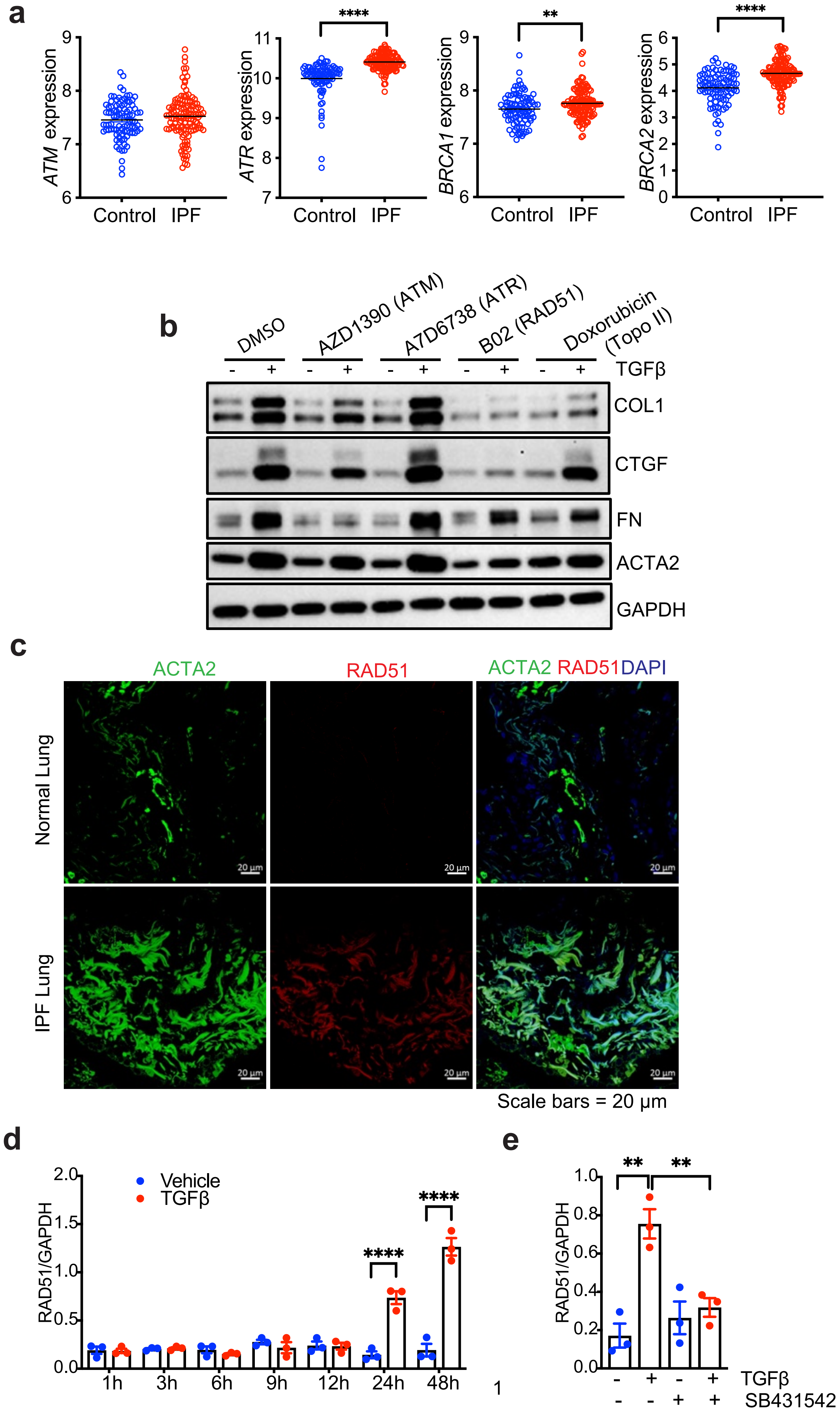

Supplementary Figure S1. Upregulation of RAD51 in pulmonary fibrosis and quantitation of TGFβ induced RAD51 accumulation. (**a**) *ATM, ATR, BRCA1, BRCA2* gene expression from 122 IPF and 91 controls patients of Lung Genomics Research Consortium (LGRC) microarray dataset (accession number GSE47460). (**b**) Quiescent NHLF cell lines were treated with either DMSO or ATM inhibitor (AZD1390), ATR inhibitor (A7D6738), RAD51 inhibitor (B02), Topoisomerase II inhibitor (Doxorubicin) in the absence (−) or presence (+) of TGFβ for 48 h and western blotted for the indicated proteins. GAPDH was used as a loading control. (**c**) Localization of RAD51 was identified using immunofluorescence staining in lung tissue sections obtained from both control and IPF patients, utilizing antibodies for ACTA2 (green), RAD51 (red), and DAPI for nuclei (blue). Co-localization can be seen in the merged images (far right side) for lung tissues from both non-IPF and IPF patients. Scale bars= 20 μm. **(d, e)** Ratios of RAD51 to GAPDH in NHLF cells were processed as depicted in Figures 1j and 1k. n = 3 independent experiments. Differences between groups were determined by two-way (d) or one-way (a, e) ANOVA test with Tukey post-hoc analysis using GraphPad Prism 10.6 software. $^{**}P < 0.01$, $^{****}P < 0.0001$.

Supplementary Figure S2

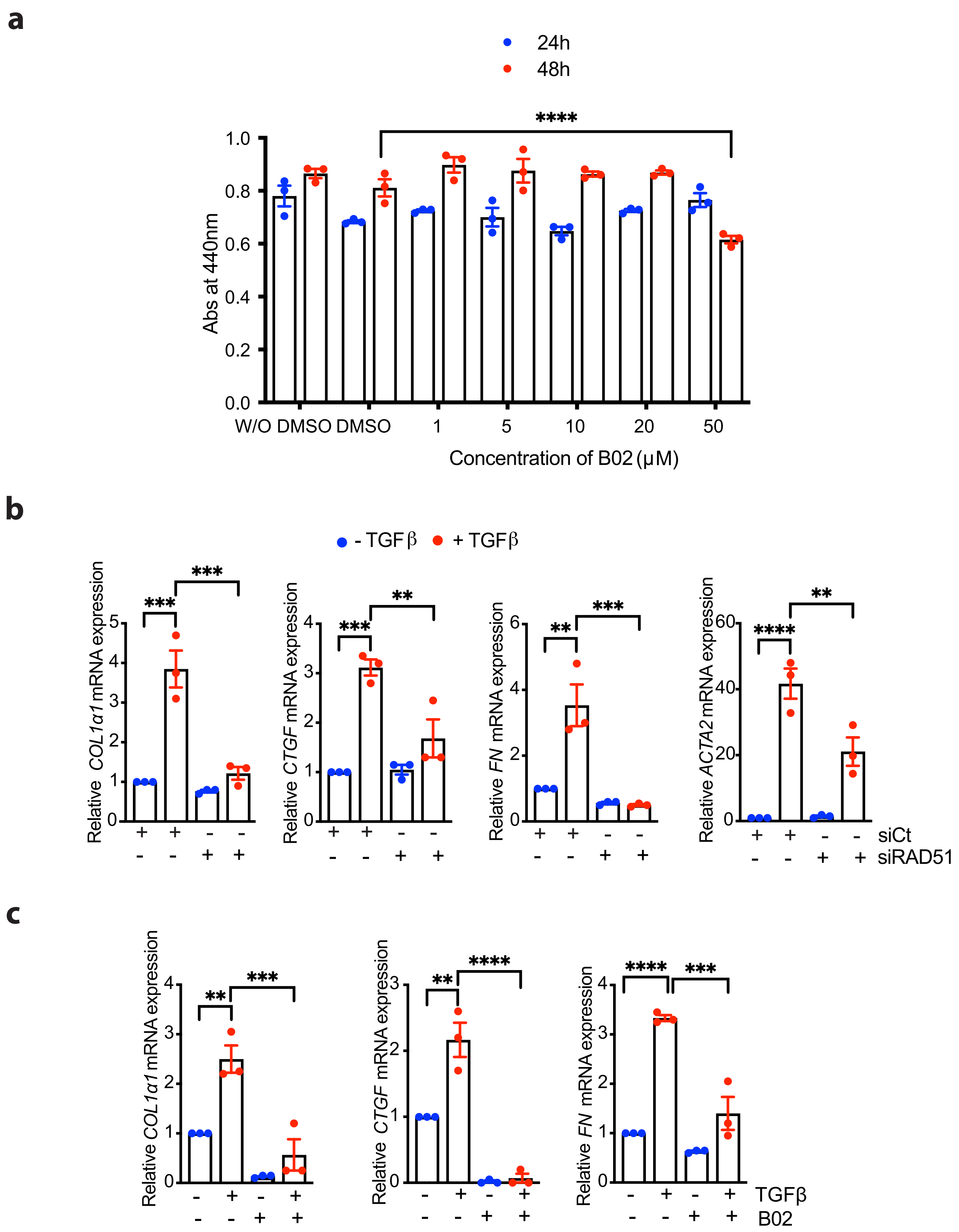

Supplementary Figure S2. Profibrotic TGFβ signaling is dependent on RAD51. (**a**) The WST-8 cell proliferation assay showed the maintained viability of NHLF cells following 24 h or 48 h incubation with RAD51 specific inhibitor B02 (1μM, 5μM, 10μM, 20μM and 50μM). 100μl/ml WST-8 reagent was added to each well containing NHLF and incubated for additional 2 h. Absorbance was measured at 440 nm using a microplate reader. Wells containing culture medium and WST-8 but without cells served as background controls. (**b**) NHLF cells were transfected with either a non-targeting control (siCt) or an siRNA targeting RAD51 (siRAD51). Following transfection, cells were treated with either a vehicle (-) or TGFβ (+) at a final concentration of 5 ng/ml. After 48 h of incubation, qPCR analyses were performed to measure the expression levels of indicated profibrotic molecules. (**c**) Quiescent NHLF cells were pretreated for 1 h with either 0.1% DMSO or 10 μM B02 (a RAD51-specific inhibitor) before the addition of vehicle or TGF-β (5 ng/ml). After a 48 h incubation period, qPCR analysis was performed to measure the expression levels of the specified profibrotic genes. n = 3 independent experiments. Differences between groups were determined by one-way ANOVA test with Tukey post-hoc analysis using GraphPad Prism 10.6 software. **P < 0.01, ***P < 0.001, ****P < 0.0001.

Supplementary Figure S3

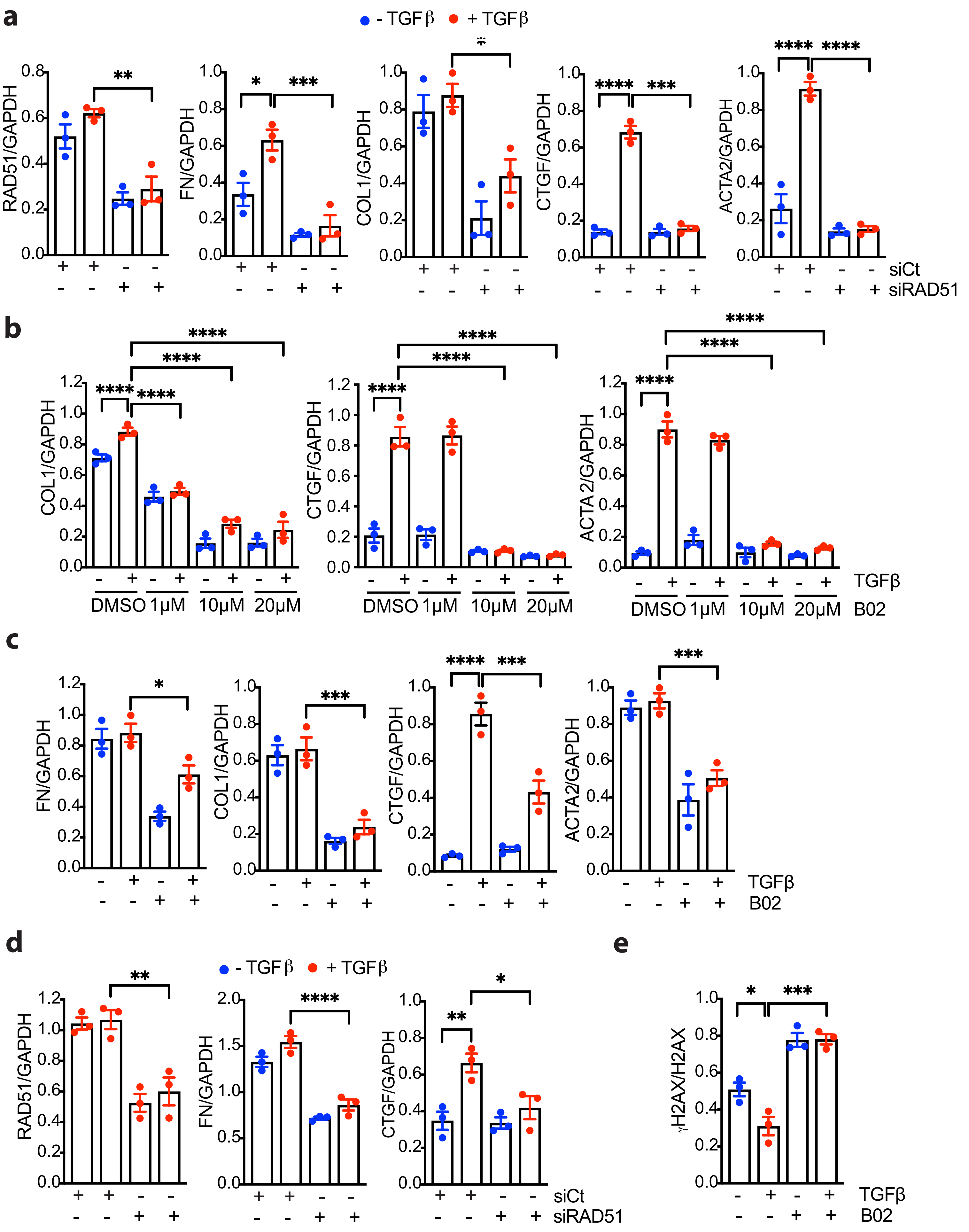

Supplementary Figure S3. Quantification of RAD51 and profibrotic markers in NHLF or IPF fibroblasts following RAD51 knockdown or inhibition. (a-d) Ratios of RAD51 and profibrotic proteins to GAPDH processed as in Fig. 2a, 2b, 2c and 2d. **(e)** Ratios of γH2AX to H2AX processed as Fig. 2m. n = 3 independent experiments. All Data reflect the means ± SEM. Differences between groups were evaluated by one-way ANOVA with Tukey post-hoc analysis using GraphPad Prism 10.6 software. *P < 0.05, **P < 0.01, ***P < 0.001, ****P < 0.0001.

Supplementary Figure S4

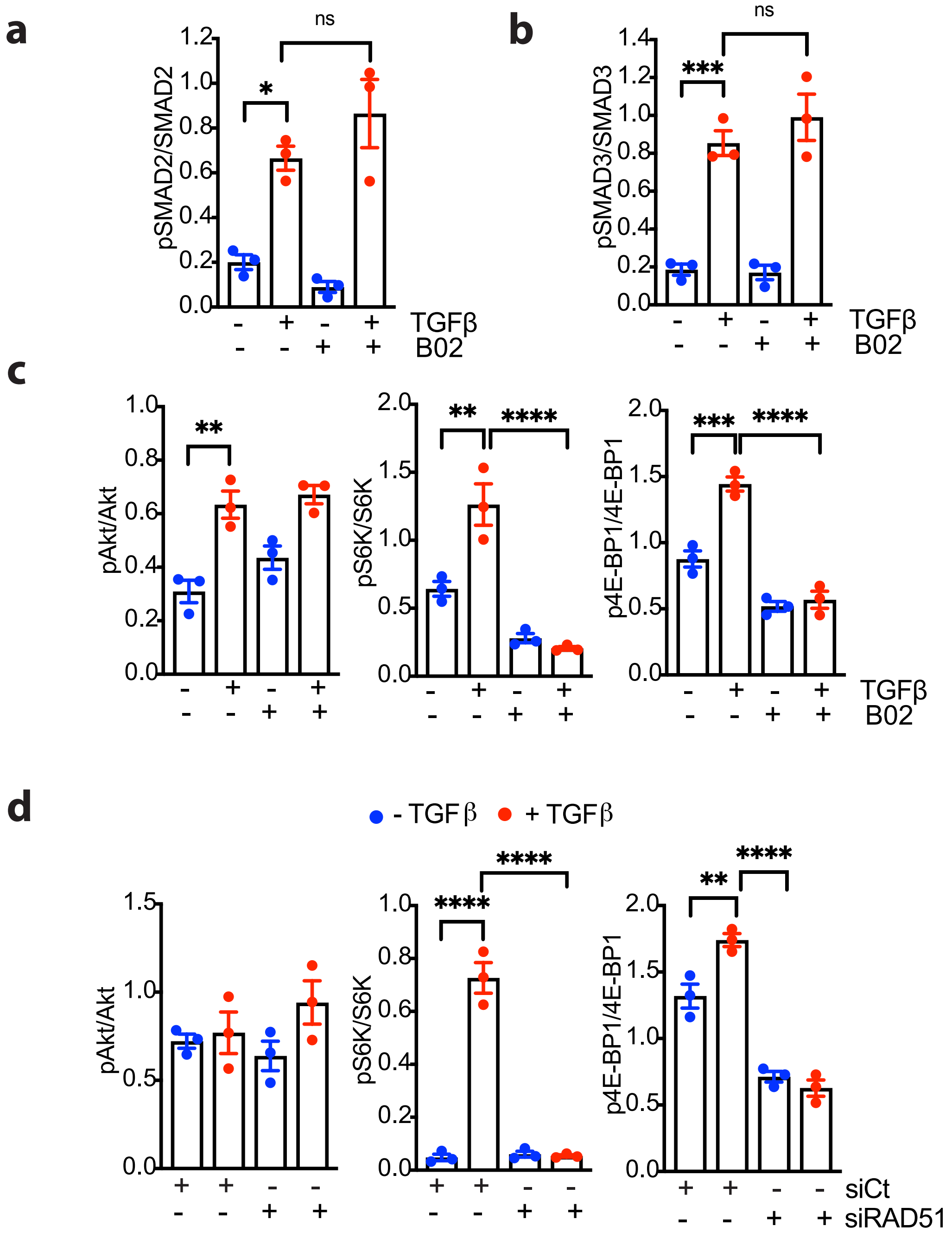

Supplementary Figure S4. Role of RAD51 in SMAD and mTOR signaling. (a, b) Ratios of pSMAD2 to SMAD2 (a) or pSMAD3 to SMAD3 (b) processed as in Fig. 3c. **(c, d)** Abundances of pS6K, pAkt and p4E-BP1 relative to total S6K, Akt or 4E-BP1, respectively as in Figure 3d and e. n = 3 independent experiments. *P < 0.05, **P < 0.01, ***P < 0.001, ****P < 0.0001.

Supplementary Figure S5

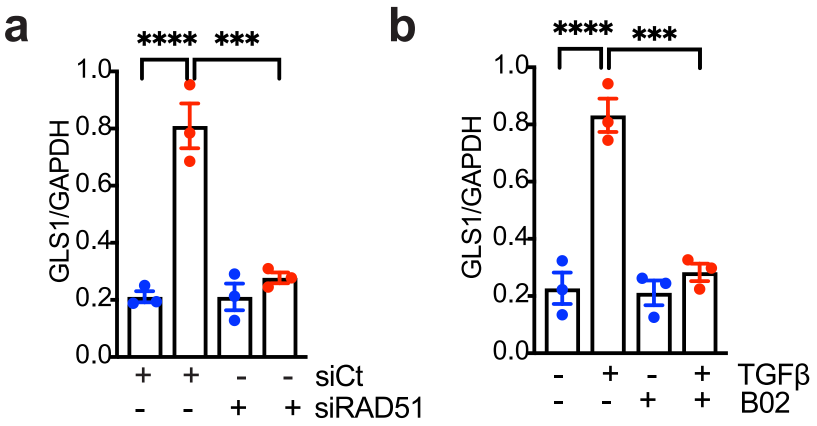

Supplementary Figure S5. Quantification of metabolic target in NHLF. (a, b) Quantification of GLS1 to GAPDH in NHLF cells processed as in Fig. 4j and k. n = 3 experiments. All Data reflect the means ± SEM. Differences between groups were evaluated by one-way ANOVA with Tukey post-hoc analysis. ***P < 0.001, ****P < 0.0001.

Supplementary Figure S6

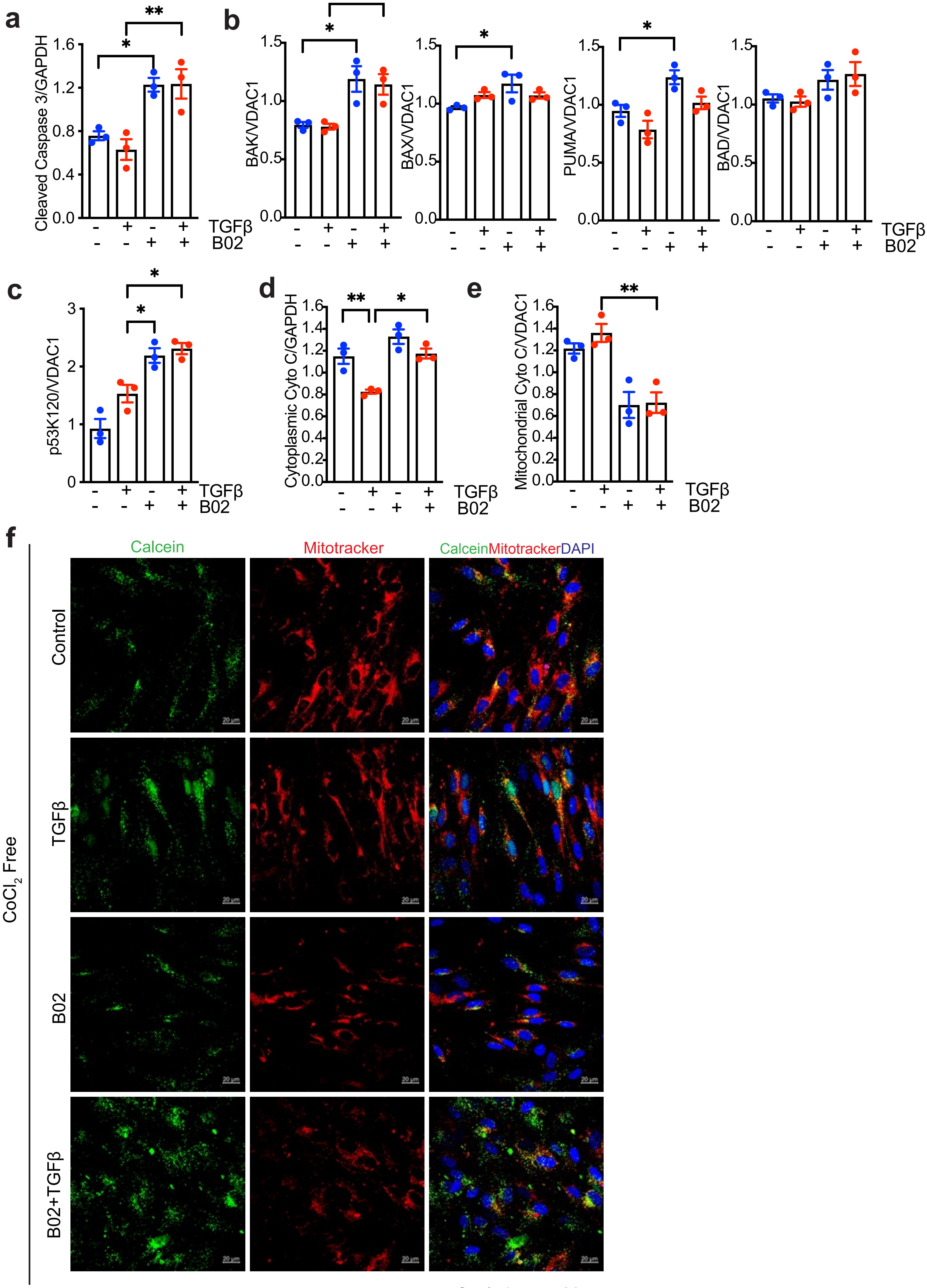

Scale bars = 20 µm

Supplementary Figure S6. Quantitation of apoptosis markers in NHLF cells after RAD51 inhibition. (a-c). Ratios of cleaved caspase 3 to GAPDH (a), proapoptotic factors to VDAC1 (b) and p53K120 to VDAC1 (c) as of Fig. 5c, f and g (mitochondrial fraction). (**d, e**) Cytochrome c to GAPDH in cytosolic fraction (d) and cytochrome c to VDAC1 in mitochondrial fraction (e), as mentioned in Fig. 5j and k. **(f)** Confocal microscopy of calcein and mitotracker was performed on TGFβ activated NHLF +/- B02 after incubation with calcein-AM (1μM) without the presence of $CoCl_2$. All Data reflect the means ± SEM. Differences between groups were evaluated by one-way ANOVA with Tukey post-hoc analysis. *$P < 0.05$, **$P < 0.01$.

Supplementary Figure S7

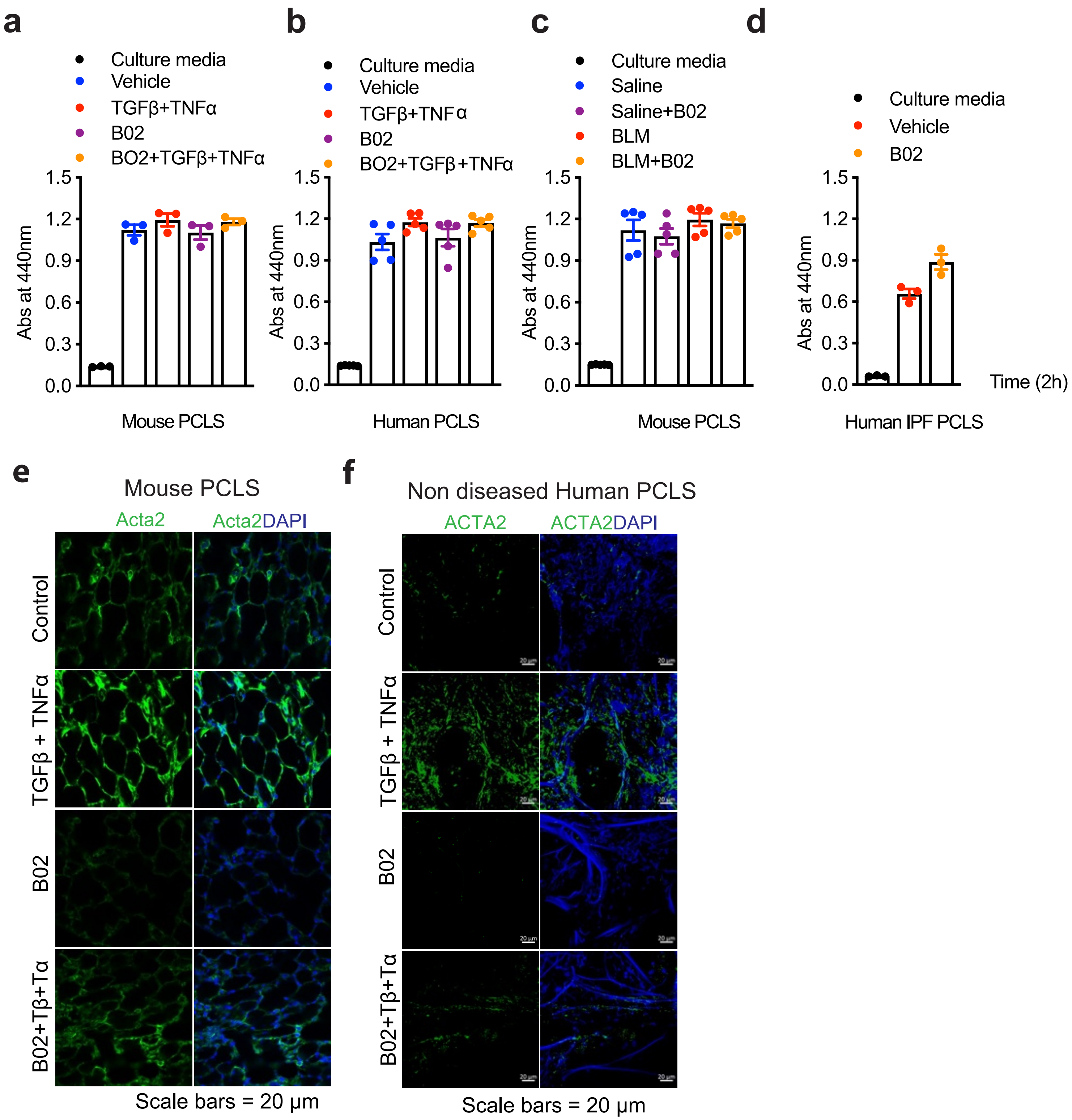

Supplementary Figure S7. Quantification of viability and profibrotic markers in mouse and human PCLS treated with B02. (a-d) Wst-8 assay showed the maintained viability of mouse PCLS (a, c) or human PCLS (b, d). Following 72 h incubation with indicated reagents, 100μl/ml WST-8 reagent was added to each well containing PCLS and incubated for 2 h. Absorbance was measured at 440 nm using a microplate reader. Wells containing culture medium and WST-8 but without slices served as background controls. **(e, f)** 8–12-week C57BL/6 mice lung (e) or adult non-diseased human lung tissue samples (f) were inflated and sliced into sections 250 μm thick using a vibrotome. These sections were then cultured in neurobasal media for 24 h, followed by treatment with 10 μM B02, 10 ng/ml TGFβ, and TNFα for an additional 72 h. Subsequently, immunofluorescence staining for the fibrotic marker ACTA2 was conducted, with green representing ACTA2 and blue representing DAPI. Data are representative of 3 independent experiments.

Supplementary Figure S8

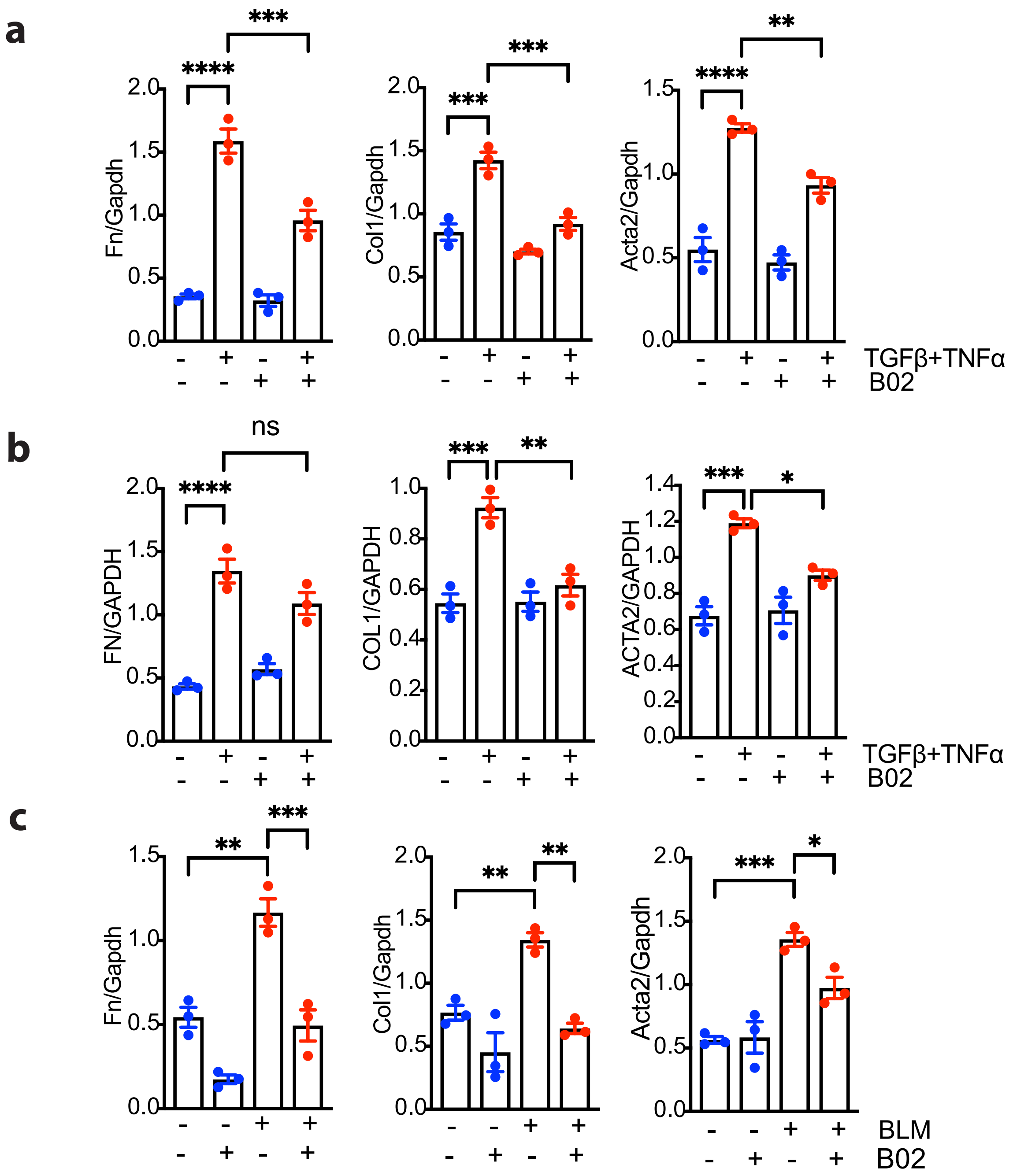

Supplementary Figure S8. Quantitation of profibrotic markers in PCLS after RAD51 inhibition. (a, b, c) Ratios of profibrotic marker (FN, Col1, ACTA2) to GAPDH processed as in Fig. 6f, g and h. n = 3 independent experiments. All Data reflect the means ± SEM. Differences between groups were evaluated by one-way ANOVA with Tukey post-hoc analysis. *P < 0.05, **P < 0.01, ***P < 0.001, ****P < 0.0001.

Supplementary Figure S9

a

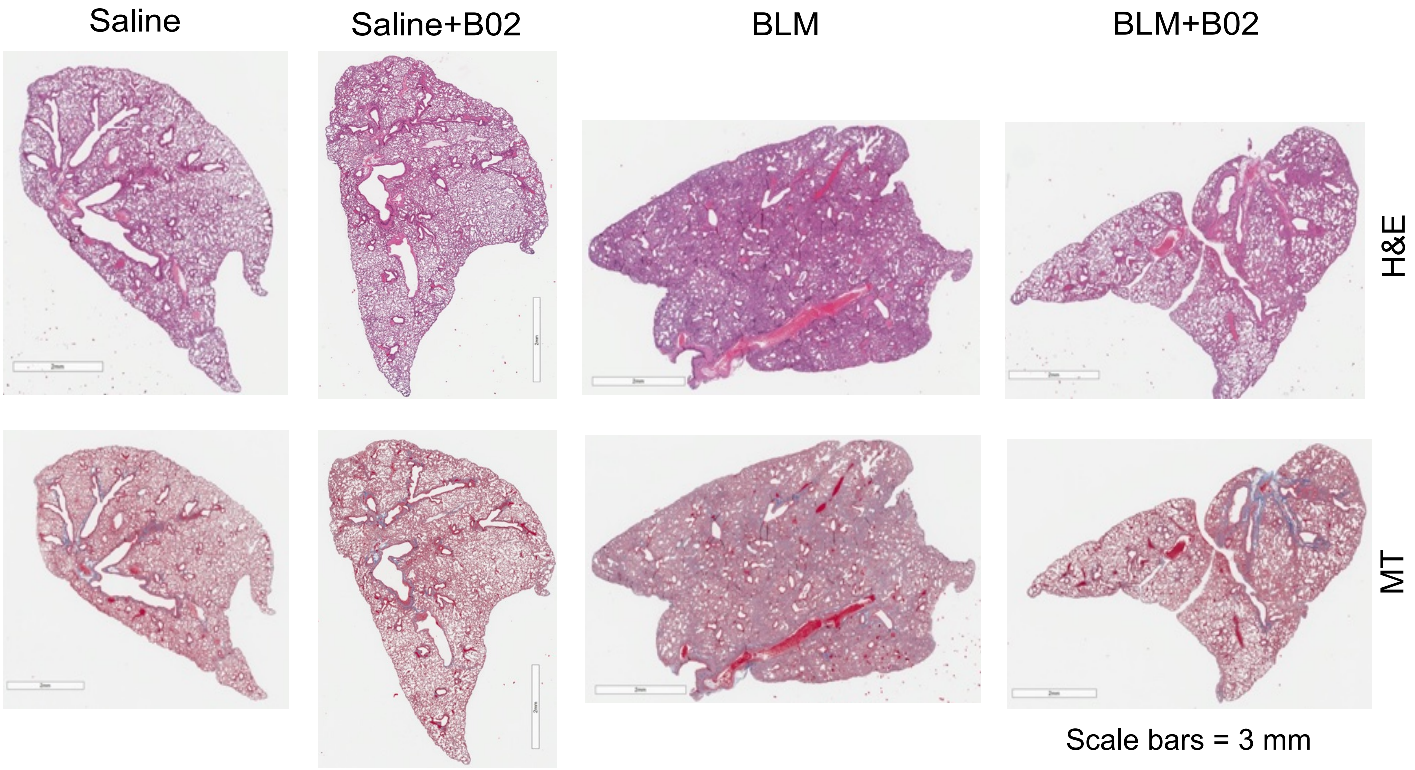

b

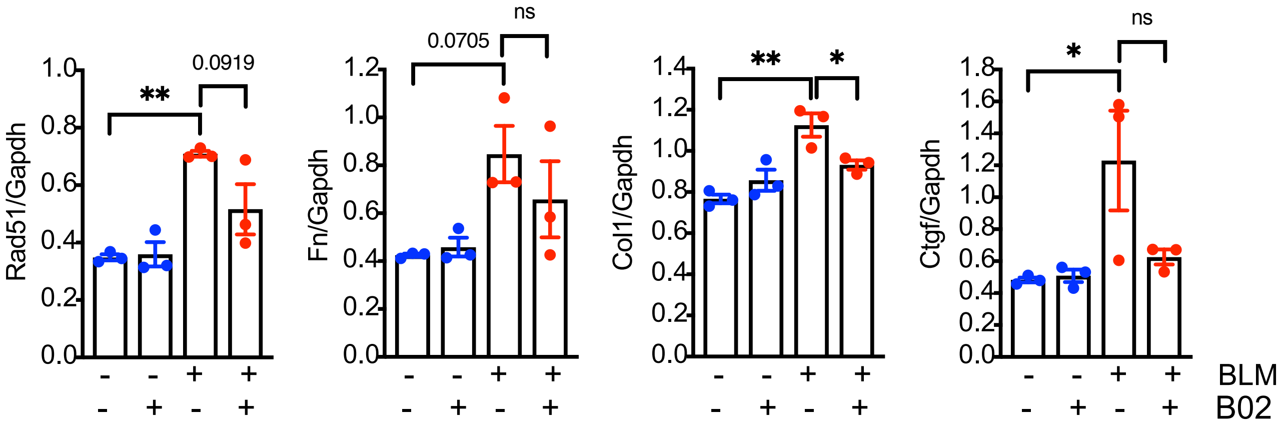

Supplementary Figure S9. B02 attenuates bleomycin-induced pulmonary fibrosis affecting lung structure. **(a)** Mice were treated as depicted in Fig. 7a, and Hematoxylin and Eosin (H&E) staining for histological analysis along with Masson’s trichrome (MT) staining for collagen deposition (indicated in blue) were conducted following euthanasia on day 23. Representative images from 6-7 mice are presented. Scale bars, 3 mm. **(b)** Quantification of Rad51, Fn Col1, and Ctgf relative to Gapdh was processed as shown in Fig. 7h. The data are represented as mean ± SEM with n = 3. Differences among groups were assessed using one-way ANOVA with Tukey post-hoc analysis. *P < 0.05, **P < 0.01.

Supplementary Figure S10

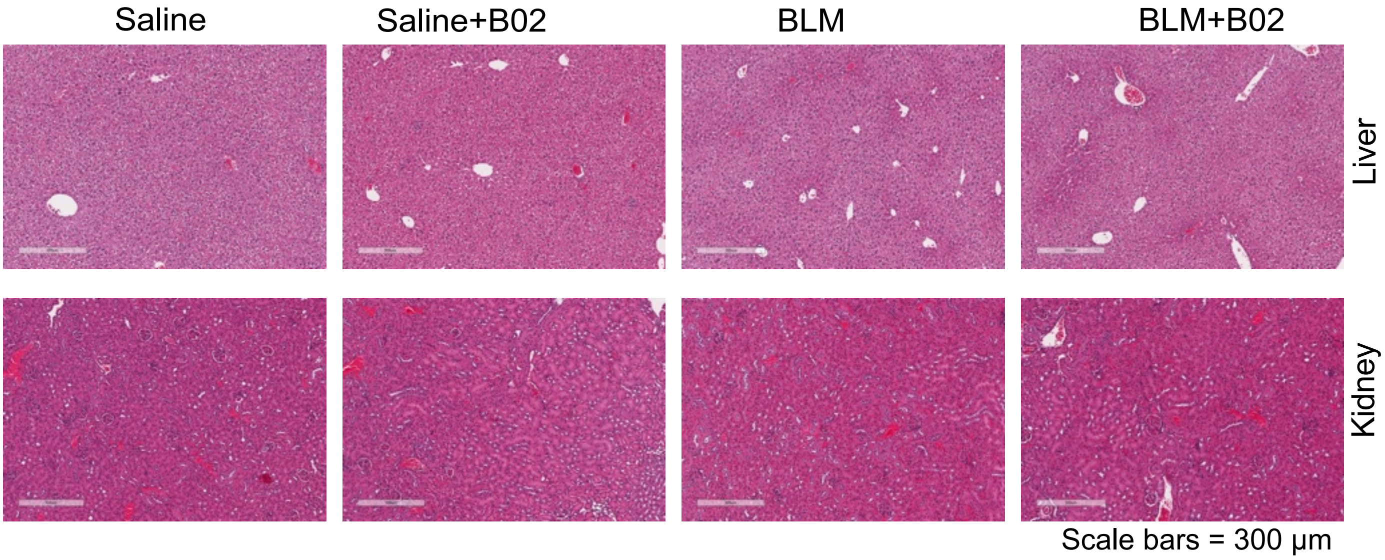

Supplementary Figure S10. Pathology of the liver and kidneys was comparable in mice that were subjected to treatment with either B02 or the vehicle. H&E staining was performed on the histology of the liver and kidney. Data are presented as of n = 6 mice.

Supplementary Figure S11

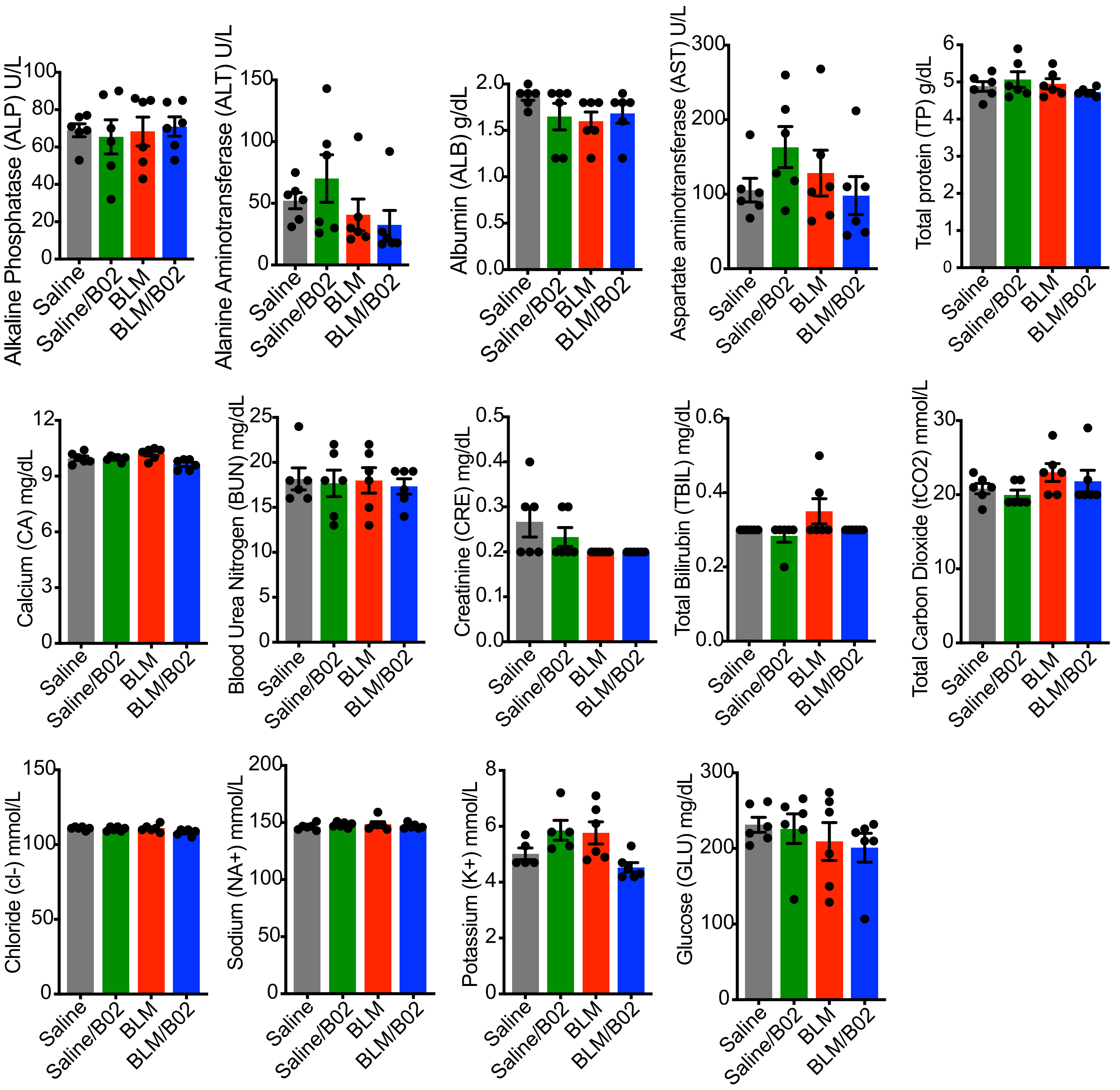

Supplementary Figure S11. Rad51 inhibition does not exhibit any demonstrable impact on the liver or kidney of mice. Male and female C57BL/6 mice were treated according to the procedures described in the Methods and in Figure 7a. Blood samples were collected on day 23 from the facial vein of unanesthetized animals using lithium heparin tubes. The serum concentrations of the specified parameters were analyzed with a Piccolo Xpress Chemistry Analyzer. Data are presented as mean ± SEM of n = 6.

Supplementary Figure S12

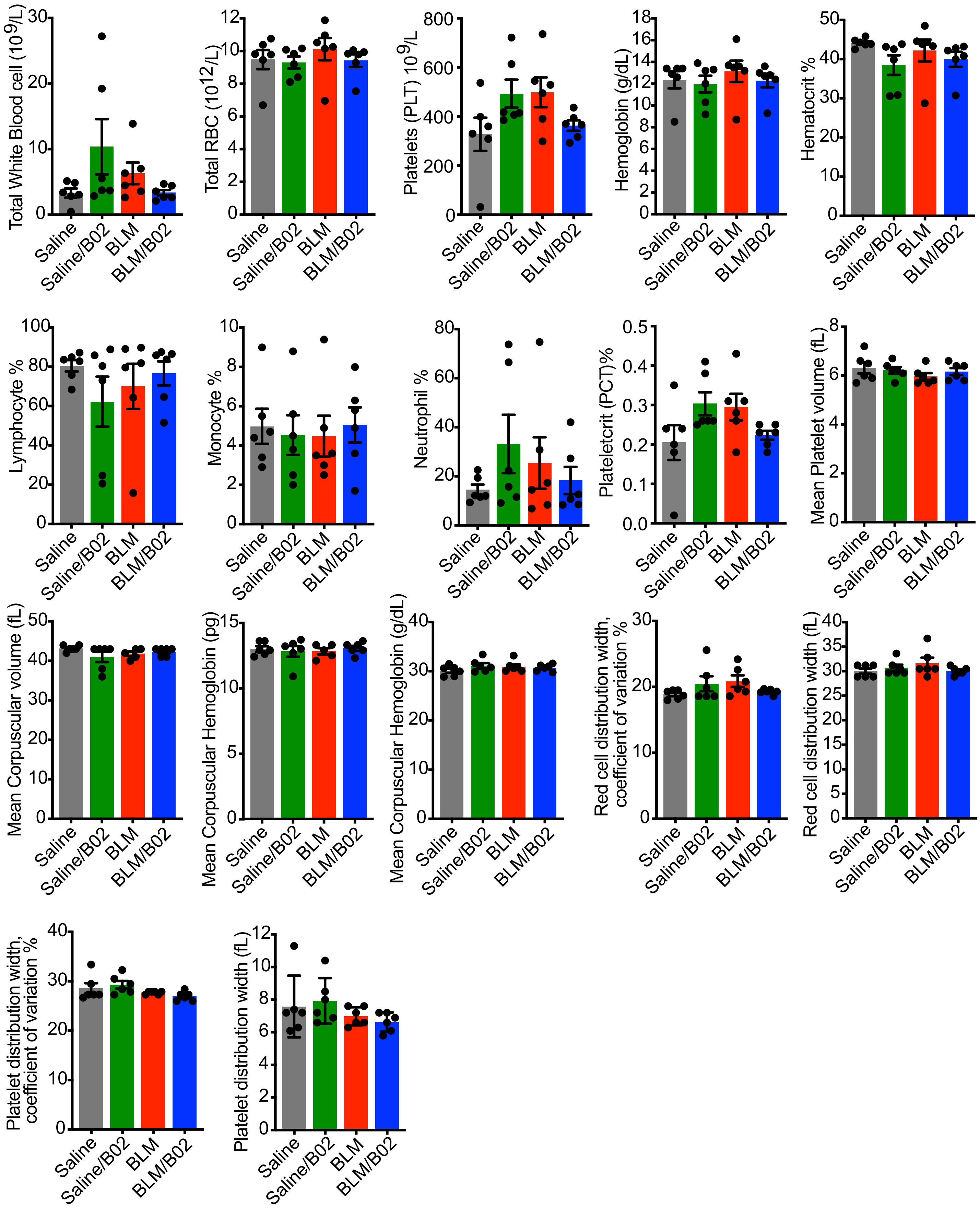

Supplementary Figure S12. B02 treatment does not impair the recruitment of inflammatory cells. C57BL/6 mice were treated as described in Methods and Figure 7a. On day 23, blood samples were collected in EDTA. Quantification of inflammatory cells and additional blood parameters was performed utilizing a VetScan HM5 Analyzer. Data are presented as mean ± SEM for n = 6.

Table S1

REAGENT or RESOURCE	SOURCE	IDENTIFIER
**Antibodies**		
Rabbit polyclonal anti-RAD51 Antibody	ThermoFisher	Cat# PA5-27195
Cleaved Caspase-3	Cell Signaling Technology	Cat# 9661S
Cytochrome c	Cell Signaling Technology	Cat# 4272S
VDAC	Cell Signaling Technology	Cat# 4866S
Donkey anti-goat IgG	Santa Cruz Biotechnology	Cat# sc-2020
Goat anti-Rabbit IgG	Jackson ImmunoResearch	Cat# 111-035-003
Goat anti-mouse IgG	Jackson ImmunoResearch	Cat# 115-035-003
PUMA	ABclonal	Cat# A17138
BAD	Cell Signaling Technology	Cat# 9292S
Glutaminase (GLS)	Novus Biologicals	Cat# NBP2-29940
Histone H2A.X	Cell Signaling Technology	Cat# 2595S
Phospho-Histone H2A.X	Cell Signaling Technology	Cat# 9718S
Mouse monoclonal Anti- GAPDH antibody	Millipore	Cat# MAB374
Goat polyclonal anti-Type I Collagen antibody	Southern Biotech	Cat# 1310-01
Goat anti-mouse IgG (Alexa Fluor 488)	abcam	Cat# ab150113
Goat anti-Rabbit IgG (Alexa Fluor 488)	abcam	Cat# ab150081
Goat anti-Rabbit IgG (Alexa Fluor 594)	abcam	Cat# ab150080
Donkey anti-Goat IgG (Alexa Fluor 488)	ThermoFisher	Cat# A-11055
Anti-BAK antibody	abcam	Cat# ab32371
Anti-BAX antibody	abcam	Cat# ab53154
Anti-p53 (acetyl K120) antibody	abcam	Cat#AB78316
Anti-alpha smooth muscle Actin antibody	abcam	Cat# ab7817
Anti-alpha smooth muscle Actin antibody	abcam	Cat# ab124964
CTHRC1	Santa Cruz Biotechnology	Cat# sc-293270
Rabbit monoclonal anti-CTGF antibody	Cell Signaling Technology	Cat# 86641S
Rabbit polyclonal anti-phospho-SMAD3 antibody	[1]	N/A
Rabbit monoclonal anti-SMAD3 antibody	Abcam	Cat# ab40854
Mouse monoclonal anti-αSMA antibody	Sigma-Aldrich	Cat# A5228
Rabbit polyclonal anti-Fibronectin antibody	Sigma-Aldrich	Cat# F3648
Rabbit polyclonal anti-SMAD2 antibody	Abcam	Cat# ab63576
Rabbit polyclonal anti-phospho-Smad2 antibody	[1]	N/A
Rabbit polyclonal anti-Akt antibody	Cell Signaling Technology	Cat# 9272
Rabbit polyclonal anti-phospho-Akt (Ser473) antibody	Cell Signaling Technology	Cat# 9271
Rabbit polyclonal anti-p70 S6 Kinase antibody	Cell Signaling Technology	Cat# 9202
Rabbit polyclonal anti-phospho-p70 S6 Kinase (Thr389) antibody	Cell Signaling Technology	Cat# 9205
Rabbit monoclonal anti-Phospho-4E-BP1 antibody	Cell Signaling Technology	Cat# 2855S
Rabbit monoclonal anti-4E-BP1 antibody	Cell Signaling Technology	Cat# 9644S
**Biological Samples**		
Normal and IPF lung fibroblast	This manuscript and [2]	U of Pittsburgh IRB# 970946, WashU IRB #201103213
**Chemicals, Peptides, and Recombinant Proteins**		
DMEM (1X) + GlutaMAX	Gibco	Cat# 10569044
B-27 Supplement (50X), serum free	Gibco	Cat# 17504044

Normal Donkey Serum	SouthernBiotech	Cat# 0030-01
CTS Neurobasal Medium	Gibco	Cat# A1371201
Hibernate-A Medium	Gibco	Cat# A1247501
Seahorse XF DMEM assay medium	Agilent technologies	Cat# 103680-100
Seahorse XF base medium	Agilent technologies	Cat# 103335-100
UltraPure Low Melting Point Agarose	Invitrogen	Cat# 16520100
Krebs-Ringer Solution, bicarbonate-buffered	Thermo Scientific	Cat# J67591.AP
Fetal Bovine Serum	HyClone Laboratories	Cat# SH3007103
Penicillin-Streptomycin	Life Technologies	Cat# 15070063
Glutamine	Agilent	Cat# 103579-100
Protease Inhibitor Cocktail	Roche	Cat# 11836153001
Recombinant Human TGFβ	R&D Systems	Cat# 240-B
Recombinant mouse TNFα	Biolegend	Cat# 575202
Recombinant human TNFα	Biolegend	Cat# 570102
LY294002	Sigma-Aldrich	Cat# L9908
MK2206	Selleckchem	Cat# S1078
Rapamycin	Selleckchem	Cat# S1039
SB431542	Tocris	Cat# 161410
Lipofectamine 3000	Thermo Fisher	Cat# L3000001
U0126	Promega	Cat# V1121
B02	Selleckchem	Cat# S8434
Dimethyl sulfoxide	Sigma-Aldrich	Cat# D8418
Phosphate Buffered Saline	Fisher bioreagents	Cat# BP399-1
Bleomycin	Hikma	Cat# NDC 00143-9240-01
**Critical Commercial Assays**		
Glutamine Colorimetric Assay Kit	BioVision	Cat# K556
RNeasy Plus Mini Kit	Qiagen	Cat# 74136
PicoProbe Lactate Assay Kit	BioVision	Cat# K638-100
Maxima Reverse Transcriptase	Life Technologies	Cat#EP0741
Hydroxyproline Assay Kit	Sigma-Aldrich	Cat#MAK008
Agilent Seahorse XF Cell Mito Stress Test Kit	Agilent Technologies	Cat#103015-100
Agilent Seahorse XF Glycolysis Stress Test Kit	Agilent Technologies	Cat#103020-100
ATP Colorimetric/Fluorometric Assay Kit	BioVision	Cat# K354
NADP/NADPH Quantitation Kit	BioVision	Cat# K347-100
Mitochondrial Transition Pore Assay Kit	Invitrogen	Cat# I35103
WST-8 Assay Kit (Cell Proliferation)	abcam	Cat#ab65475
**Oligonucleotides**		
siRNA targeting human RAD51	Santa Cruz Biotechnology	Cat# sc-36361
siRNA targeting human SMAD2	Santa Cruz Biotechnology	Cat# sc-38374
siRNA targeting human SMAD3	Santa Cruz Biotechnology	Cat# sc-38376
Control siRNA	Santa Cruz Biotechnology	Cat#sc-37007
Primers for all quantitative reverse transcription polymerase chain reaction (RT-qPCR), please refer to the Table S2	This paper	N/A
**Software and Algorithms**		
ImageJ	[3]	https://imagej.nih.gov/ij/
GraphPad Prism 10.6		https://www.graphpad.com/scientific-software/prism/
BioRender		https://biorender.com

Table S2.

Primer for human qRT-PCR

Gene	Forward	Reverse
RAD51	CAACCCATTTCACGGTTAGAGC	TTCTTTGGCGCATAGGCAACA
COL1A1	GAGGGCCAAGACGAAGACATC	CAGATCACGTCATCGCACAAC
CTGF	GTCCAGCACGAGGCTCA	TCGCCTTCGTGGTCCTC
FN	TGTCAGTCAAAGCAAGCCCG	TTAGGACGCTCATAAGTGTCACCC
ACTA2	GACAATGGCTCTGGGCTCTGTAA	CTGTGCTTCGTCACCCACGTA
TBP	GCCCGAAACGCCGAATATAATC	GTCTGGACTGTTCTTCACTCTTGG

Primer for mouse qRT-PCR

Gene	Forward	Reverse
Col1a1	ATCTCCTGGTGCTGATGGAC	ACCTTGTTTGCCAGGTTCAC
Ctgf	CACAGAGTGGAGCGCCTGTTC	GATGCACTTTTTGCCCTTCTTAATG
Fn	TGACAACTGCCGTAGACCTG	ATCTAGCGGCATGAAGCACT
Tbp	GAAGTTCCCTATAAGGCTGGAAG	AGGAGAACAATTCTGGGTTTGA

References:

1. Wilkes MC, Murphy SJ, Garamszegi N, Leof EB. Cell-type-specific activation of PAK2 by transforming growth factor beta independent of Smad2 and Smad3. *Mol Cell Biol* 2003; **23**(23): 8878-89.

2. Pilewski JM, Liu L, Henry AC, Knauer AV, Feghali-Bostwick CA. Insulin-like growth factor binding proteins 3 and 5 are overexpressed in idiopathic pulmonary fibrosis and contribute to extracellular matrix deposition. *The American journal of pathology* 2005; **166**(2): 399-407.

3. Schneider CA, Rasband WS, Eliceiri KW. NIH Image to ImageJ: 25 years of image analysis. *Nature methods* 2012; **9**(7): 671-5.
